# Zinc finger proteins in cancer progression

**DOI:** 10.1186/s12929-016-0269-9

**Published:** 2016-07-13

**Authors:** Jayu Jen, Yi-Ching Wang

**Affiliations:** Department of Pharmacology, College of Medicine, National Cheng Kung University, No.1, University Road, Tainan, 70101 Taiwan, Republic of China; Department of Basic Medical Sciences, College of Medicine, National Cheng Kung University, No.1, University Road, Tainan, 70101 Taiwan, Republic of China

**Keywords:** Zinc finger protein, Transcription factor, Cancer progression

## Abstract

Zinc finger proteins are the largest transcription factor family in human genome. The diverse combinations and functions of zinc finger motifs make zinc finger proteins versatile in biological processes, including development, differentiation, metabolism and autophagy. Over the last few decades, increasing evidence reveals the potential roles of zinc finger proteins in cancer progression. However, the underlying mechanisms of zinc finger proteins in cancer progression vary in different cancer types and even in the same cancer type under different types of stress. Here, we discuss general mechanisms of zinc finger proteins in transcription regulation and summarize recent studies on zinc finger proteins in cancer progression. In this review, we also emphasize the importance of further investigations in elucidating the underlying mechanisms of zinc finger proteins in cancer progression.

## Background

Transcription factors play a central role in regulating gene expression, and therefore coordinate a plethora of biological processes, including differentiation, development, metabolism, apoptosis, autophagy and stemness maintenance [1–5]. Based on different DNA binding motifs, transcription factors can be majorly categorized into classical zinc fingers [6], homeodomains [7], and basic helix-loop-helix [8]. Among these, classical zinc finger containing proteins (ZNFs) form the largest family of sequence-specific DNA binding protein, which are encoded by 2 % of human genes [9, 10]. To date, 8 different classes of zinc finger motifs have been reported, including Cys2His2 (C2H2) like, Gag knuckle, Treble clef, Zinc ribbon, Zn2/Cys6, TAZ2 domain like, Zinc binding loops and Metallothionein [11]. Different types of zinc finger motifs show great diversity of biological functions. Notably, in addition to DNA binding, studies have recently revealed the RNA, protein and lipids interacting abilities of zinc finger motifs [12–15]. Therefore, with different combinations of multiple zinc finger motifs, ZNFs can greatly expand their diverse role in gene regulations under different cell contexts or stimuli. The general mechanism of gene regulation by ZNFs and their great variety of roles in cancer progression will be discussed in this review.

### The transcription regulation of ZNFs

C2H2-type zinc finger motif is the largest group of all zinc finger motif classes. According to the InterPro database (updated on April 14th 2016), there are 5,926 members in the C2H2-type ZNF family. C2H2-type zinc finger motif is composed of CX2CX3FX5LX2HX3H, and its two cysteine and two histidine residues fold into a finger-like structure of a two-stranded antiparallel β-sheet and an α-helix after interacting with zinc ions [16, 17]. Studies have demonstrated that two to three successive C2H2-type zinc finger motifs are the most suitable unit for DNA binding [6]. In addition, GC-rich or GT-rich sequences serve as C2H2-type ZNF *cis*-regulatory elements. For example, CTGGCAGCGC has been revealed as SP1 consensus binding element to transcriptionally activate BRK1 expression, while (T/A)(G/A)CAGAA(T/G/C) is the consensus element for ZNF217 to suppress E-cadherin expression [18, 19].

In addition to tandem zinc finger motifs, C2H2-type ZNF also contains other functional domains, such as BTB (Broad-Complex, Tramtrack, and Bric-a-brac)/POZ (poxvirus and zinc finger), the Krüppel-associated box (KRAB), and SCAN (SRE-ZBP, CTfin51, AW-1 and Number 18 cDNA) domain. These functional domains may control subcellular localization, DNA binding and gene expression by regulating selective binding of the transcription factors with each other or with other cellular component. For instance, zinc finger protein GATA-1 has been reported to interact with different partners, including Fli-1, Sp1, EKLF and PU.1 [20–22].

ZNF proteins can carry out different functions with different partners and even elicit opposing actions on different partners. For example, physical interaction between GATA-1 and Fli-1, a member of Ets family of transcriptional activator, cooperatively activate the expression of megakaryocyte-specific genes, including GPIX and GPIbalpha, at transcriptional level [20]. In contrast, interacting with PU.1, another Ets family member, blocks GATA-1 DNA binding ability and therefore inhibits erythroid differentiation [23]. Recent study also shows that ZEB1, a transcription repressor of differentiation-associated genes, turns its function into a transcriptional co-activator of a common ZEB1/YAP target genes through interacting with YAP and therefore leading to aggressive cancer phenotype [24].

Studies have demonstrated that ZNF proteins show diverse regulation mechanisms on a wide variety of downstream genes through recruiting different chromatin modifiers. Some ZNF proteins work as transcriptional repressors by recruiting co-repressors [25–27]. For example, ZNF217 has been found to suppress downstream gene expression by interacting with co-repressors, including CoREST, lysine demethylase 1, histone deacetylase 2 and C-terminal binding protein [25]. Some ZNF proteins, on the other hand, work as transcriptional activators by interacting with co-activators, including CBP/p300 and C/EBP [28, 29]. These studies clearly indicate that *trans*-acting proteins play important roles in determining ZNFs as transcription activators or repressors.

### Post-translational modifications on ZNFs

The post-translational modifications (PTMs) of ZNFs, especially acetylation and phosphorylation, add another layer of regulation for ZNFs in which transcription may be activated or repressed. GATA1, a transcription factor that contains 2 highly conserved zinc finger motifs, is found acetylated at the lysine residues adjacent to the C terminal zinc finger by acetyltransferase CBP and p300. Acetylation of GATA1 shows stable association with chromatin probably by facilitating protein interactions, such as bromodomain-containing protein Brd3 [30–32]. Erythroid Krüppel-like factor, also known as EKLF, is acetylated at lysine residues 288 and 302 near its zinc finger motif mediated by CBP and p300 [33]. The acetylated EKLF at lysine residue 288 can transactivate β-globin expression through recruiting the large erythroid complex (ERC-1) that contains SWI/SNF chromatin-remodeling proteins and histone 3.3 [33, 34]. Another C2H2 zinc finger protein, YY1, is acetylated by p300/CBP associated factor (PCAF) at its zinc finger motif and inhibits its DNA binding capacity. Acetylation mediated by p300 and PCAF at the central glycine-lysine rich domain of YY1, however, does not affect DNA binding affinity but fully suppresses target gene transcription [35].

Phosphorylation on serine or threonine residues of the ZNFs linker peptide has been reported [36]. ZNFs, including Ikaros, Sp1 and YY1, are found to be highly phosphorylated on threonine/serine residues of their linker peptide during mitosis and therefore abrogated their DNA binding ability [37, 38]. Rizkallah et al. generated an antibody raised against phosphorylated linker peptide TGEKP to show that about 50 % of all linkers in 80 % of C2H2-type ZNFs are phosphorylated, indicating that phosphorylation is a highly coordinated mechanism to keep ZNFs away from DNA during mitosis [39].

### The oncogenic ZNFs in cancer progression

Recent studies revealed that aberrant expression of C2H2 ZNF proteins contributes to tumorigenesis in different aspects (summarized in Table 1). For example, amplification and overexpression of ZKSCAN3, also known as ZNF306 or ZNF309, was first reported in invasive colorectal cancers. The authors showed that ZKSCAN3 knockdown in colorectal cancer cells inhibited anchorage-independent growth and orthotopic tumor growth, while ZKSCAN3 overexpression exerted opposite effects [40]. To identify ZKSCAN3 downstream genes, these authors further conducted expression array and identified candidate target genes enriched in growth, cell migration, angiogenesis and proteolysis [41]. Studies confirm that ZKSCAN3 transcriptionally activates integrin β4 and vascular endothelial growth factor, which are involved in ZKSCAN3-mediated colorectal tumorigenesis [41]. In addition, ZKSCAN3 is also found to be amplified and overexpressed in multiple myeloma and prostate cancer [42, 43]. The overexpression of ZKSCAN3 enhances cell proliferation through transcriptionally activating cyclin D2 expression [42]. Interestingly, a recent study reveals a novel role of ZKSCAN3 in autophagy using cervical cancer, colon cancer, neuroblastoma, and ovarian cancer models [4]. Chauhan et al., show that ZKSCAN3 translocates into the nucleus and acts as a master transcriptional repressor of a large set of genes involved in autophagy and lysosome biogenesis, including *Map1lC3b* and *Wipi2*, under serum stimulation [4].

**Table 1:** Summary of differential roles of ZNF proteins in cancer progression

ZNFs	Aliases	Role	Cancer models	Target genes	Mechanism in tumorigenesis	References
ZKSCAN3	ZNF306, ZNF309	Oncogene	Colorectal cancer	Integrin β4 ↑, VEGF ↑	Promotes cancer cell growth, migration, angiogenesis, proteolysis	[40, 41]
			Multiple myeloma	Cyclin D2 ↑	Enhances cell proliferation	[42]
			Prostate cancer	-	Promotes cell migration	[43]
			Cervical, colon, ovarian cancer, neuroblastoma	MAP1LC3B ↓, WIPI2 ↓	Suppresses autophagy and lysosome biogenesis	[4]
ZNF322A	ZNF388, ZNF489	Oncogene	Lung cancer	-	Chromosome locus 6p22.1 is amplified	[44]
				ADD1 ↑, CCND1 ↑, p53 ↓	Promotes cell growth, migration and invasion	[45]
ZNF304	-	Oncogene	Colorectal	p14^ARF^ ↓, p15^INK4B^ ↓, p16^INK4A^ ↓	Suppresses tumor suppressor genes through recruiting a co-repressor complex, including DNMT1	[48]
			Ovarian cancer	Integrin β1 ↑	Activates Src/focal adhesion kinase and paxillin and therefore prevents anoikis	[49]
ZNF139	ZKSCAN1, ZNF36, ZSCAN33, KOX18	Oncogene	Gastric cancer	-	Serves an independent prognostic factor for gastric cancer patients	[50]
				Survivin ↑, x-IAP ↑, Bcl2 ↑, Caspase-3 ↓, Bax ↓	Promotes cell proliferation and inhibits apoptosis	[51]
				MMP-2 ↑, MMP-9 ↑, ICAM-1 ↑, TIMP-1 ↓	Promotes cell migration and invasion	[52]
				MDR-1/P-gp ↑, MRP1 ↑, Bcl-2 ↑, Bax ↓	Contributes to multi-drug resistance	[53]
ZFX	ZNF926	Oncogene	Hepatocellular carcinoma	Nanog ↑, SOX2 ↑	Confers self-renewal properties and chemoresistance	[5]
			Nasopharyngeal carcinoma	E-cadherin ↓	May be involved in EMT	[54]
			Glioma, lung, oral, breast cancer	-	Promotes cell proliferation and survival	[55, 56, 58, 60]
			Gastric cancer	-	Promotes cell growth through up-regulating ERK-MAPK pathway	[57]
			Gallbladder cancer	-	Promotes proliferation, migration and invasion potentially through activation of PI3K/AKT pathway	[59]
			Glioblastoma	c-Myc ↑	Promotes glioma stem cell maintenance	[61]
ZEB1	ZFHX1A, DELTAEF1	Oncogene	Breast cancer	ESRP2 ↓	Promotes TGF-β-induced EMT	[68]
			Glioma	-	SHP-2 up-regulates ZEB1 expression to mediate EMT, invasion and growth	[69]
			Cervix, breast cancer, osteosarcoma, adrenal carcinoma	E-cadherin ↓	Down-regulates E-cadherin and cell polarity factors by recruiting co-repressor CtBP or BRG1	[70, 71]
			Cervix, colorectal cancer	-	Activates genes involved in TGF-β/BMP signaling by recruiting p300 and P/CAF	[72, 73]
			Lung cancer	E-cadhein ↑, ST14 ↑, Vimentin ↑	Confers EMT-related acquired resistance to EGFR-TKI	[74]
			Breast cancer	VEGFA ↑	Promotes angiogenesis	[75]
ZNF545	ZFP82	TSG	Nasopharyngeal, esophageal, lung, gastric, colon, breast cancer	-	Induces cell apoptosis by repressing ribosome biogenesis and NF-kB and AP-1 signaling	[76]
ZNF331	ZNF361, ZNF463	TSG	Gastric cancer	DSTN ↓, EIF5A ↓, GARS ↓, DDX5 ↓, STAM ↓, UQCRFS1 ↓, SET ↓, ACTR3 ↓	Inhibits cell growth, migration and invasion	[78]
			Gastrointestinal, esophageal cancer	-	Promoter hypermethylation is found in various cancer types	[79, 80]
ZNF24	ZNF191, Kox17	TSG	Breast cancer	VEGF ↓	Inhibits angiogenesis	[82, 83]
			Gastric cancer	-	miR940 promotes cancer migration and invasion by targeting ZNF24	[84]
ZNF668	-	TSG	Breast cancer	-	Suppresses cell proliferation by promoting MDM2 autoubiquitination and therefore p53 stabilization	[85]
			Osteosarcoma	-	Involved in DNA repair by regulating chromatin relaxation and recruiting repair proteins to DNA lesions	[86]
ZHX1	-	TSG	Gastric cancer	-	miR-199a-3p promotes cell proliferation and suppresses apoptosis by targeting to ZHX1	[88]
				CCND1 ↓, CCNE ↓, Bcl2 ↓, Bax ↑, cleaved Caspase-3 ↑	Induce G1/S arrest and apoptosis	[89]
ZNF395	PBF, HDBP2	Oncogene	Ewing’s sarcoma, osteosarcoma, renal cell carcinoma	-	Overexpressed in various cancers	[90–92]
			Glioblastoma	-	Induced under hypoxia stress	[93]
			Skin and cervix cancer, glioblastoma	IFIT1/ISG56 ↑, IFI44 ↑, IFI16 ↑	Supports inflammation and cancer progression	[94]
		TSG	Liver cancer	-	miR-525-3p promotes cell migration and invasion by targeting ZNF395	[95]
Kaiso	ZNF348, ZBTB33	TSG	Breast and colon cancer	CCND1 ↓	Suppresses cell proliferation	[99]
		Oncogene	Breast cancer	Vimentin ↑, Slug ↑, ZEB1 ↑	Involved in TGF-β-mediated metastasis	[100]
			Prostate cancer	miR-31 ↓	Promotes cell migration and invasion	[101]
			Breast and colorectal cancer	HIF-1α ↓	-	[102]

-, target not-determined

ZNF322A, also known as ZNF388 or ZNF489, consists of 11 tandem repeats of C2H2 zinc finger motif. ZNF322A was first identified as oncogene by Lo et al., showing that *ZNF322A* residing region is amplified in both Asian and Caucasian lung cancer patients [44]. Further study reveals that ZNF322A promotes cell proliferation, migration and invasion through transcriptionally activating cyclin D1 and alpha-adducin but suppressing p53 in lung cancers [45]. Multivariate Cox regression analysis indicates ZNF322A is an independent risk factor of poor outcome in lung cancer patients [45]. Notably, ZNF322A mouse ortholog, Zfp322a, is reported as a novel essential component of the transcription network, which maintains the self-renewal and pluripotency of mouse embryonic stem (mES) cells [46]. Zfp322a promotes OKSM (Oct4, Klf4, Sox2, c-Myc)-induced mouse embryonic fibroblast reprogramming to mES cells by transcriptionally activating Oct4 and Nanog expression [46]. The study on Zfp322a implies a potential role of human ZNF322A in maintaining the pluripotency of embryonic stem cells or cancer stem cells.

ZNF304, which contains a KRAB domain and 13 C2H2 zinc finger motifs, was first identified by AU-motif directed display and RACE in 2002 [47]. ZNF304 plays a pivotal role in silencing tumor suppressors, including p14^ARF^, p15^INK4B^ and p16^INK4A^, through recruiting a co-repressor complex that includes DNA methyltransferase DNMT1 [48]. In addition, an integrative bioinformatic analysis of The Cancer Genome Atlas ovarian cancer dataset and experimental validation reveals the association between ZNF304 and ovarian cancer metastasis [49]. The authors show that ZNF304 transcriptionally activates integrin β1 expression, which subsequently activates Src/focal adhesion kinase and paxillin and eventually prevents anoikis [49]. Using delivery of ZNF304 siRNA by a dual assembly nanoparticle, these authors successfully conducted a sustained ZNF304 silencing which increased anoikis and reduced ovarian tumor growth in orthotopic mouse models [49].

ZNF139 is significantly overexpressed in gastric cancer patients. Cox survival analysis reveals ZNF139 overexpression as an independent prognostic factor for gastric cancer patients [50]. ZNF139 has been reported to promote proliferation and inhibit apoptosis through up-regulating the expression of Survivin, x-IAP and Bcl-2, and down-regulating Caspase-3 and Bax [51]. In addition, ZNF139 promotes cancer migration and invasion in gastric cancer by increasing the expression of MMP-2, MMP-9 and ICAM-1, and decreasing the expression of TIMP-1 [52]. ZNF139 also contributes to multi-drug resistance by enhancing the expression of MDR-1/P-gp, MRP1, Bcl-2 while inhibiting Bax expression [53].

Overexpression of zinc finger protein, X-linked (ZFX) has been shown to promote cell growth and metastasis in laryngeal squamous cell carcinoma, glioma, non-small cell lung cancer, gastric cancer, oral squamous cell carcinoma, gallbladder cancer and breast cancer [5, 54–60]. In addition, ZFX is found to confer self-renewal properties and chemoresistance in hepatocellular carcinoma through transcriptional activation of Nanog and SOX2 expression [5]. Fang et al. also showed that ZFX transcriptionally up-regulates c-Myc expression leading to glioma stem cell maintenance [61]. Inhibition of ZFX using siRNA oligo or drug treatment suppresses cancer progression, indicating the potential of oncogenic ZNFs as therapeutic targets [62, 63].

Zinc finger E-box-binding homeobox, ZEB1, is a well-studied transcription factor involved in Epithelial-Mesenchymal Transition (EMT) in several cancer types, including breast cancer, lung cancer, pancreatic cancer and prostate cancer [64–67]. ZEB1 expression in cancer cells is elevated upon signaling induction, including TGF-β and platelet-driven growth factor receptor-α signaling [68, 69]. As an activator of EMT, increased ZEB1 binds to E-boxes containing downstream targets, including E-cadherin and cell polarity factors, and represses their transcription by recruiting co-repressors CtBP or SWI/SNF chromatin-remodeling protein BRG1 [70, 71]. Notably, studies also reveal that ZEB1 can transcriptionally activate genes involved in TGF-β/BMP signaling through recruiting co-activators, p300 and P/CAF [72, 73]. In addition to its role in EMT, ZEB1 overexpression further contributes to EMT-related acquired resistance to epidermal growth factor receptor-tyrosine kinase inhibitors (EGFR-TKI) in non-small cell lung cancer through transcriptionally up-regulating E-cadherin, ST14 and vimentin [74]. Moreover, Yoshida et al., show that silencing ZEB1 expression restores sensitivity to EGFR-TKI, suggesting targeting ZEB1 could be a potential therapy to resensitize TKI-resistant tumors [74]. A recent study also reveals a novel role of ZEB1 in promoting angiogenesis in breast cancer [75]. The authors show that ZEB1 overexpression in breast cancer cells recruits Sp1 to *VEGFA* promoter region and activates VEGFA expression and secretion, therefore promoting angiogenesis in vitro and in vivo [75].

### The tumor suppressor ZNFs in cancer progression

In addition to cancer promotion, several ZNFs have been found to function as tumor suppressors. For example, ZNF545, which is down-regulated in cancer cells as a consequence of promoter methylation, acts as a tumor suppressor by inducing cell apoptosis, repressing ribosome biogenesis and suppressing NF-kB and AP-1 signaling in nasopharyngeal, esophageal, lung, gastric, colon and breast cancer [76]. Notably, methylated degrees of five CpG sites (-232, -214, -176, -144 and -116) discriminate gastric cancer patients’ survival outcome with higher CpG methylation predicting poorer overall survival [77]. Another ZNF known to be inactivated by promoter hypermethylation is ZNF331, also known as ZNF361 or ZNF463 [78–80]. Overexpression of ZNF331 inhibits cell growth by down-regulating genes, including DSTN, EIF5A, GARS, DDX5, STAM, UQCRFS1 and SET, and inhibits cell migration/invasion by down-regulating genes, including DSTN and ACTR3 [78].

ZNF24, also known as ZNF191 or Kox17, contains 4 Krüppel-like C2H2 zinc finger domains on C-terminus that function as DNA binding domains [81]. ZNF24 suppresses VEGF expression by binding to the proximal *VEGF* promoter, and negatively regulates tumor growth by inhibiting angiogenesis in breast cancer [82, 83]. Using transgenic zebra fish model, Jia et al., demonstrate that expression of human ZNF24 induces vascular defects, which can be recovered by VEGF overexpression [83]. Clinical studies of human breast cancer confirm the inverse correlation between ZNF24 and VEGF, indicating the tumor suppressor role of ZNF24 in breast cancer tumorigenesis by inhibiting angiogenesis [83]. Interestingly, a recent study shows that miR940 is up-regulated in gastric cancer and promotes gastric cancer migration and invasion by targeting tumor suppressor ZNF24 [84].

ZNF668 is a member of Krüppel C2H2 zinc finger protein family, which possesses 16 C2H2-type zinc fingers. ZNF668 facilitates p53 stabilization and activity by disrupting MDM2-mediated ubiquitination and degradation in breast cancer [85]. In addition, ZNF668 interacts with Tip60 to enhance H2AX hyperacetylation in response to ionizing radiation and promote RPA phosphorylation and recruitment to DNA damage foci upon UV damage, therefore leading to chromatin relaxation and loading of DNA repair proteins [86].

Zinc-fingers and homeoboxes-1 (ZHX1), which contains two C2H2 zinc finger motifs and five homeodomains, has been reported to be down-regulated in hepatocellular carcinoma and gastric cancer [87–89]. ZHX1 induces G1/S arrest through down-regulating cyclin D1 and cyclin E expression, and enhances apoptosis through down-regulating Bcl2 and up-regulating Bax and cleaved Caspase-3 [89]. Of note, Wang et al., demonstrate that miRNA, miR-199a-3p, targets ZHX1 for RNA degradation to promote cell proliferation and suppresses apoptosis in gastric cancer. Reconstitution of ZHX1 expression abrogates gastric cancer oncogenicity [88].

### ZNFs: double-edged sword in tumorigenesis

Some ZNFs have been shown to play different roles in different cancer types and stimuli. For example, ZNF395 is overexpressed in various cancers, including Ewing sarcomas, osteosarcomas and renal cells carcinomas [90–92]. Moreover, ZNF395 expression is induced under hypoxic stress in glioblastoma, neuroblastoma and skin cancer [90, 93, 94]. Hypoxia-induced ZNF395 can transcriptionally up-regulate cancer-related genes and interferon-stimulated genes, such as IFIT1/ISG56, IFI44 and IFI16, in an IKK signaling-dependent manner [94]. These results implicate ZNF395 as a novel transcription factor which supports inflammation and cancer progression. However, a recent study reveals the tumor suppressor role of ZNF395 in liver cancer. The authors show that miR-525-3p, which is overexpressed in liver cancer, promotes liver cancer cell migration and invasion by targeting and down-regulating ZNF395 expression [95]. The clinical analysis indeed confirms the inverse correlation of miR-525-3p and ZNF395 in liver cancer [95]. These studies collectively show that ZNF395 may play different roles in different cancer types.

Kaiso, also known as ZNF348 or ZBTB33, belongs to the BTB/POZ subfamily of ZNFs. Kaiso can bind to sequence-specific or methyl-CpG DNA using its zinc finger motifs, while its N-terminus POZ domain helps homodimerization or heterodimerization with chromatin co-repressors, including nuclear receptor co-repressor I [96–98]. By recruiting chromatin co-repressors, Kaiso transcriptionally suppresses downstream gene expression. Kaiso was first identified as a tumor suppressor that transcriptionally suppressed oncogenic genes in sequence- or methyl-CpG-specific manner. For example, Kaiso represses cyclin D1 expression by binding to *CCND1* promoter in a sequence- and methyl-CpG-specific manner in breast and colon cancer [99]. Since then, more and more studies have demonstrated the oncogene role of Kaiso in various cancers. For example, Kaiso is found highly expressed in triple negative breast cancers and involved in TGF-β-mediated metastasis by up-regulating several EMT genes, including Vimentin, Slug and ZEB1 [100]. High expression of Kaiso in prostate cancer promotes cell migration and invasion through transcriptional suppression of miR-31 expression in methyl CpG-specific manner [101]. In addition, Pierre et al., show that Kaiso transcriptionally suppresses HIF-1α expression by targeting to methylated *HIF1A* promoter in breast and colorectal cancer [102]. Kaiso is a versatile ZNF, which exerts different functions in different cell types in respond to different stimuli.

## Conclusion

**Fig. 1:**
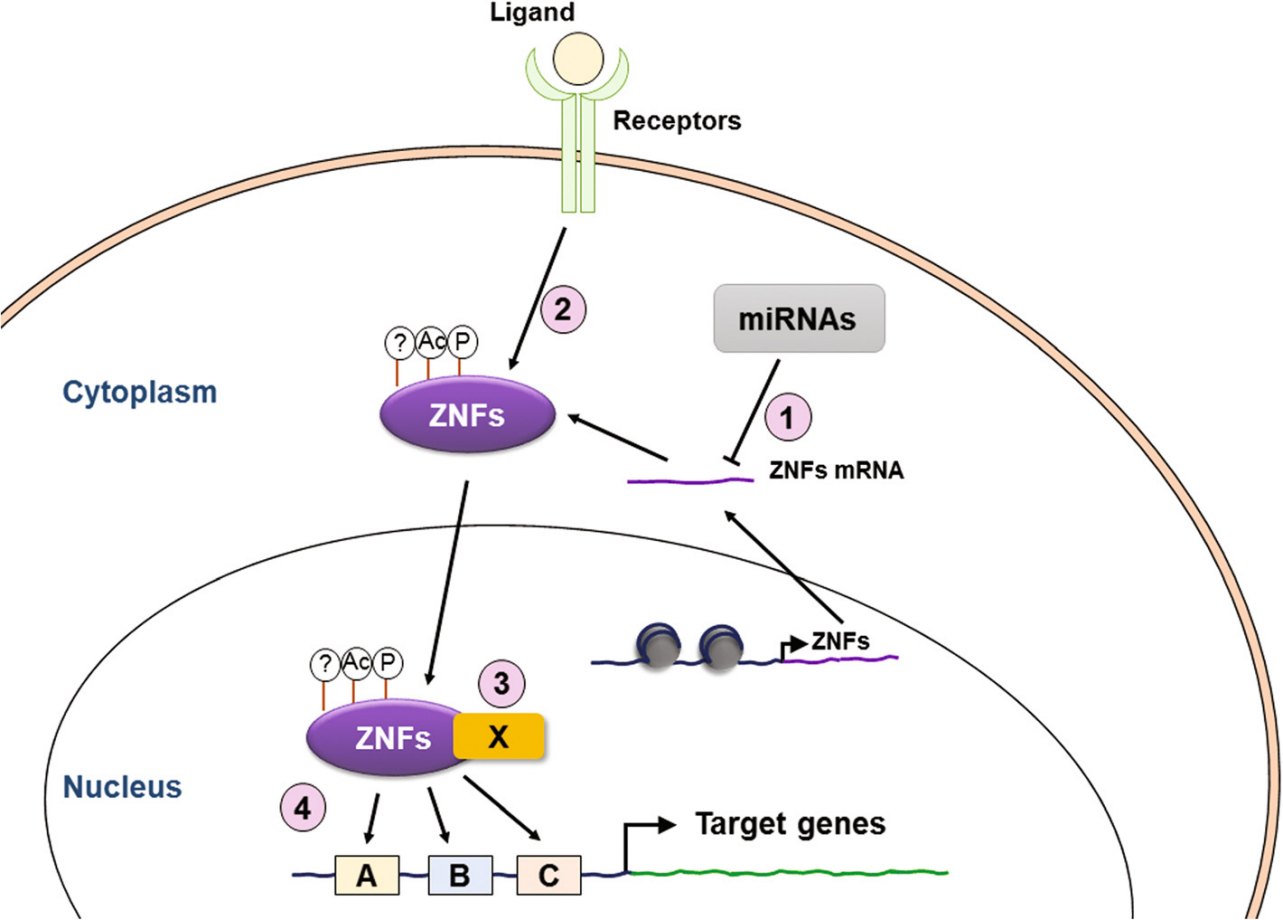
Various regulations of ZNF proteins’ functions in cancer progression. The versatile roles of ZNF proteins in cancer progression can be regulated at different levels. Differential expression of ZNF proteins in different cancer types can be regulated by 1) cancer-related miRNAs, including miR-199a-3p, miR-525-3p, miR-940 and miR-31, or 2) different environmental stimuli, which activate signaling cascades and therefore fine-tune ZNF protein functions through various of PTMs, including phosphorylation (P) and acetylation (Ac). 3) ZNF proteins at different protein domains or with various PTMs recruit different interacting proteins namely X, including transcription co-activators/co-repressors, chromatin modifiers and other transcription factors, to activate or suppress downstream genes. 4) ZNF proteins show diverse sequence-specific DNA binding abilities due to different combinations of zinc finger motifs shown as boxes

Recent studies show that C2H2 ZNF proteins play important roles in cancer progression through regulating transcription of downstream genes, which are involved in proliferation, apoptosis, migration and invasion. Although more and more studies have been focused on the underlying mechanism of C2H2 ZNF transcription regulation, results remain conflicting. It is now understood that different layers of regulations lead C2H2 ZNF proteins to different roles in tumorigenesis. In this review, we summarize various levels of ZNF proteins regulation in tumorigenesis (Fig 1). First, differential expression levels of ZNF proteins in different cancer types are regulated by cancer-related miRNA, including miR-199a-3p, miR-525-3p, miR-940 and miR-31. Second, different environmental stimuli activate signaling cascades and therefore fine-tune ZNF protein functions through various PTMs, including phosphorylation and acetylation. PTMs regulation affects DNA binding abilities and interacting proteins recruitments of ZNF proteins. Third, ZNF proteins at different protein domains or with various PTMs recruit different interacting proteins, including transcription co-activators/co-repressors, chromatin modifiers and other transcription factors. Therefore, ZNF proteins can activate or suppress downstream genes by recruiting different interacting partners. Fourth, ZNF proteins show diverse sequence-specific DNA binding abilities with different combinations of zinc finger motifs. Knowing the complexities and diversities of ZNF proteins, it is important to elucidate the underlying mechanisms of C2H2 ZNF proteins in different cancers under different environmental stimuli. Therefore, drugs targeting specific C2H2 ZNF protein expression or activity can be developed for therapeutic strategy against tumors in a specific stage of cancer progression.

## Abbreviations

BTB, Broad-Complex, Tramtrack, and Bric-a-brac; C2H2, Cys2His2; EGFR-TKI, epidermal growth factor receptor-tyrosine kinase inhibitors; EMT, Epithelial-Mesenchymal Transition; ERC-1, erythroid complex; KRAB, Krüppel-associated box; mES, mouse embryonic stem; PCAF, p300/CBP associated factor; POZ, poxvirus and zinc finger; PTMs, post-translational modifications; SCAN, SRE-ZBP, CTfin51, AW-1 and Number 18 cDNA; TGF-β, transforming growth factor-β; ZFX, zinc finger protein, X-linked; ZHX1, Zinc-fingers and homeoboxes-1; ZNF, Zinc finger
